# Compassionate use of tezepelumab in near fatal asthma: A case report and review of the literature

**DOI:** 10.1016/j.rmcr.2025.102223

**Published:** 2025-04-26

**Authors:** Mako Nakajima, Masashi Matsuyama, Yuki Yamazaki, Hiroya Sunabe, Yuta Takahashi, Keishun Boku, Takeshi Miura, Tomohiro Tamura, Yoshiaki Inoue, Nobuyuki Hizawa

**Affiliations:** a Department of Pulmonary Medicine, Faculty of Medicine, University of Tsukuba, Ibaraki, Japan; b Department of Emergency and Critical Care Medicine, University of Tsukuba, Ibaraki, Japan; c Respiratory Center, Ibaraki Prefectural Central Hospital, Kasama, Japan

**Keywords:** Near fatal asthma, Veno-venous extracorporeal membrane oxygenation (VV-ECMO), Biologics, Tezepelumab

## Abstract

A 38-year-old man with near fatal asthma requiring intubation and invasive mechanical ventilation (IMV) was transferred to our hospital. Veno-venous extracorporeal membrane oxygenation (VV-ECMO) was initiated due to hypotension and mediastinal emphysema secondary to barotrauma. Since high-dose systemic corticosteroid therapy failed to relieve the severe bronchospasm, a single dose of tezepelumab was administered on a compassionate basis. This intervention resulted in successful extubation 7 days later. This is a rare case in which a biologic agent was used as a rescue therapy for near fatal asthma. This case is presented along with a review of the relevant literature.

## 1 Introduction

Severe asthma exacerbations can progress to respiratory failure, respiratory acidosis, and respiratory arrest requiring invasive mechanical ventilation (IMV), a condition known as near fatal asthma [1]. Though systemic corticosteroid therapy is considered a cornerstone in the management of severe asthma exacerbations [2,3], various biologics are increasingly being used as controller medications in severe asthma [4]. Recent reports suggest that biologics may also be effective in cases of near fatal asthma [[5], [6], [7], [8], [9], [10], [11]], particularly when high-dose systemic corticosteroid therapy fails. However, the impacts of biologic agents on near fatal asthma remain uncertain due to the limited number of documented cases. The case of a 38-year-old man with near fatal asthma who failed to respond to high-dose systemic corticosteroid therapy and required IMV and veno-venous extracorporeal membrane oxygenation (VV-ECMO) is reported. The patient was subsequently treated with tezepelumab, resulting in successful extubation. To the best of our knowledge, this is the first reported case in Japan of near fatal asthma treated with tezepelumab in an intensive care unit (ICU). This case is reported along with a review of the relevant literature.

## 2 Case report and literature review

A 38-year-old man was diagnosed with asthma as a child. His asthma symptoms were mildly relieved at age 15 years, but worsened at age 17 years when he started smoking. He continued to be an active smoker. He was prescribed triple therapy (inhaled corticosteroid [ICS]/long-acting β_2_-agonist [LABA]/long-acting muscarinic antagonist [LAMA]) and a leukotriene receptor antagonist (LTRA) by a local physician, but adherence was poor. His medical history included dissociative identity disorder. Over the past year, he had presented to the emergency department (ED) at least twice for asthma exacerbations, each time refusing inpatient care. He was treated with short courses of oral corticosteroids (OCS) during these visits. The patient had documented allergies to house dust mite, pollen, and animal dander, with total IgE of 1015 IU/ml and eosinophilia (up to 1574/μl). His body mass index was 20.1 kg/m^2^, indicating that he was not obese. There was no history of atopic dermatitis or allergic rhinitis.

The patient presented to a nearby ED with wheezing, dyspnea, and chest tightness. One week prior, he had experienced worsening fatigue and insomnia due to asthma exacerbation and was using albuterol frequently. He had not received OCS therapy from his local physician during this time. On arrival, arterial blood gas analysis showed a pH of 7.19, PaCO_2_ of 85.1 mmHg, and PaO_2_ of 83.7 mmHg while receiving 6–8 L/min of oxygen by mask. Due to impaired consciousness, he was admitted to the ICU and managed with invasive mechanical ventilation (IMV). Despite 48 hours of high-dose intravenous systemic corticosteroid therapy (betamethasone 4 mg every 6 hours) and short-acting $\beta_{2}$ agonists, his peak inspiratory pressure remained high, and he developed mediastinal emphysema. To prevent further barotrauma, VV-ECMO was considered, and the patient was transferred to our hospital.

**Fig. 1:**
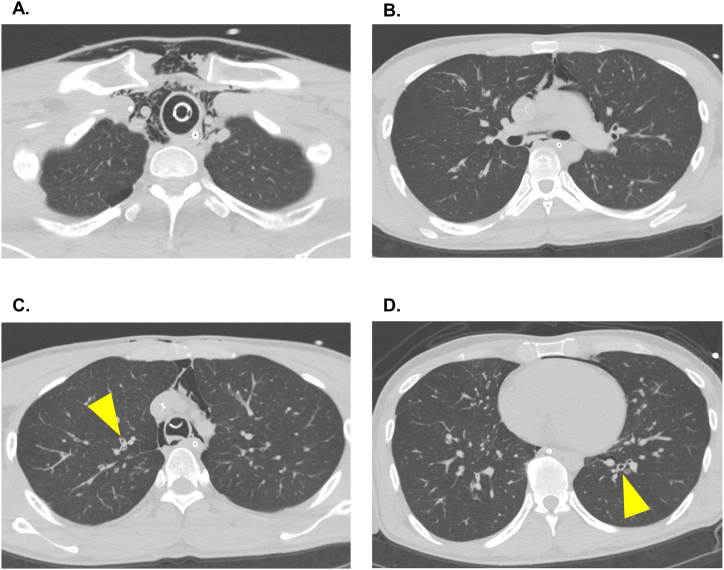
Chest computed tomography findings on admission before veno-venous extracorporeal membrane oxygenation (VV-ECMO) treatment **(A)** Mediastinal and subcutaneous emphysema due to invasive mechanical ventilation is observed. **(B)** Mediastinal emphysema due to invasive mechanical ventilation is observed. **(C), (D)** The arrow shows bronchial wall thickening.

**Fig. 2:**
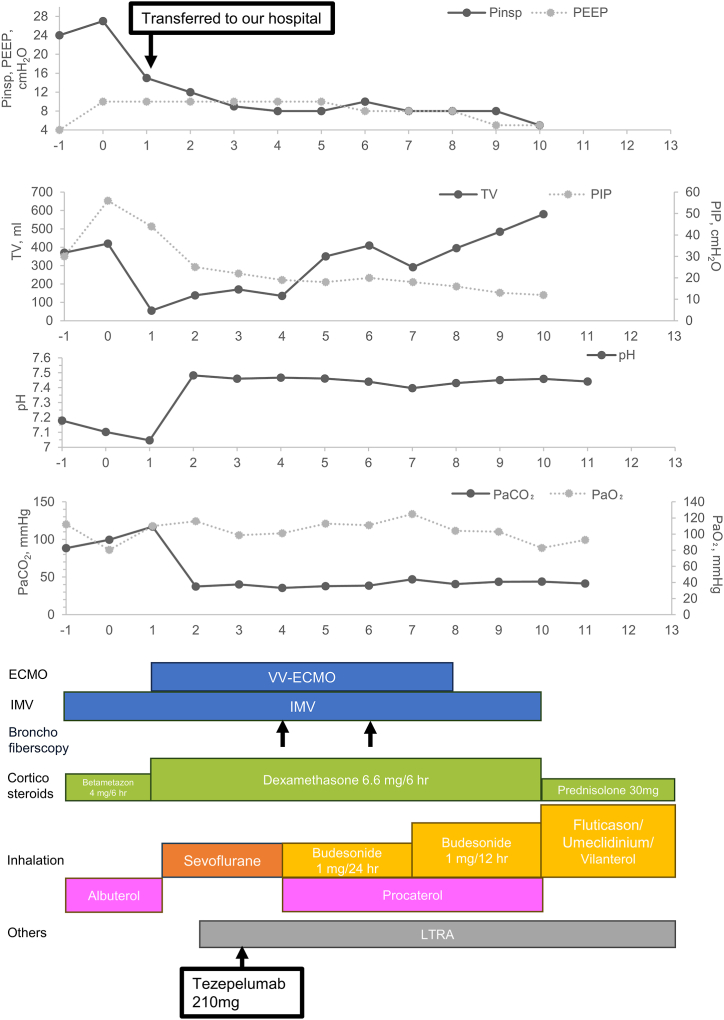
Time course of this case Abbreviations: IMV, invasive mechanical ventilation; VV-ECMO, veno-venous extracorporeal membrane oxygenation; PEEP, positive end-expiratory pressure; Pinsp, inspiratory airway pressure; LTRA, leukotriene receptor antagonist; PIP, peak inspiratory pressure; TV, tidal volume.

On admission, chest computed tomography (CT) showed mediastinal and subcutaneous emphysema, hyperinflated lung fields, and bronchial wall thickening (Fig. 1). Sinus CT showed no evidence of sinusitis, and a PCR test for COVID-19 was negative. In the ICU, the peak inspiratory pressure remained elevated (40–45 cmH_2_O), and the patient experienced profound respiratory acidosis with unstable blood pressure. V-V ECMO (HCS-MP23P, MERA, Senko Medical Instrument Mfg. Co. Ltd, Tokyo, Japan), with a 23-Fr drainage cannula from the right femoral vein (BE-PVL2355, GETINGE, Tokyo, Japan) and an 18-Fr return cannula to the right jugular vein (PCKC-A18, MERA, Senko Medical Instrument Mfg. Co. Ltd) was initiated, resulting in rapid improvement of respiratory acidosis (Fig. 2). However, tidal volume (TV) did not improve despite continued high-dose intravenous systemic corticosteroid therapy (dexamethasone 6.6 mg every 6 hours), LTRA, short-acting $\beta_{2}$ agonists, and sevoflurane inhalation (AnaConDa, Sedana Medical, Stockholm, Sweden). After 4 days of high-dose corticosteroid therapy with no improvement, compassionate use of a biologic was considered. The patient's profile included active smoking, corticosteroid resistance, and elevated serum IgE levels. In addition, his peripheral blood eosinophil count was 0/μl on admission due to corticosteroid administration. Based on these factors, tezepelumab was chosen over IL-5 pathway inhibitors (mepolizumab or benralizumab).

Airway resistance improved significantly to 20–30 cmH_2_O within 24 hours of tezepelumab administration. Due to the presence of highly viscous sputum, bronchoscopy-assisted expectoration was performed as needed. TV gradually increased after tezepelumab treatment (Fig. 2). The patient was successfully weaned from VV-ECMO 4 days after tezepelumab administration, extubated 7 days after treatment, and transferred to a general ward 8 days after treatment (Fig. 2). No tezepelumab-related side effects were observed.

## 3 Discussion

The role of biologic agents as rescue therapy during severe exacerbations remains underexplored. However, a few reports suggest that biologics can be effective in acute exacerbations outside of ICU settings [12,13]. For example, Ramakrishnan et al. described a case in which benralizumab was successfully used as an alternative to corticosteroids in a patient with a history of steroid-related adverse events [12]. This is the first reported case in Japan of near fatal asthma treated with tezepelumab in an ICU setting, managed with IMV and VV-ECMO. Although biologic agents are primarily used as long-term controller medications for severe asthma [4], emerging evidence suggests their potential utility as acute interventions in specific clinical scenarios.

Tezepelumab is the newest biologic agent for severe asthma [14]. Thymic stromal lymphopoietin (TSLP) levels are elevated in response to smoking, excessive $\beta_{2}$ -agonist use, viral infections [[15], [16], [17]], and exposure to allergens such as house dust mite. TSLP is also implicated in corticosteroid resistance [18]. Tezepelumab targets upstream inflammatory pathways in asthma and has shown efficacy across both type 2 and non-type 2 phenotypes [19]. Given the patient's smoking status, frequent use of albuterol, resistance to high-dose corticosteroid therapy, and elevated serum IgE levels, tezepelumab may have been particularly effective in this case. In addition, TSLP has been reported to directly induce airway smooth muscle contraction [20,21] and contribute to mucus production [22]. This suggests that tezepelumab may have played a significant role in reducing bronchospasm, alleviating mucoid impaction, and improving airway resistance in the present patient.

Table 1 summarizes eight cases of near fatal asthma managed in the ICU and treated with biologics, including the present case. In all cases, high-dose intravenous corticosteroid failed to relieve severe bronchospasm, necessitating the introduction of biologics. In each case, patients were weaned from VV-ECMO or extubated within one week following biologic administration. Subcutaneous biologics, such as omalizumab, mepolizumab, benralizumab, and tezepelumab generally reach peak serum levels within approximately 7 days [[23], [24], [25], [26]], which aligns with the observed timeline for clinical improvement and subsequent weaning from VV-ECMO or extubation (Table 1).

**Table 1:** Comparison of cases with near fatal asthma treated with biologics in intensive care unit (ICU) settings.

Reference (PMID)	Year	Sex	Age	ICU	Smoking	Time of asthma onset	Biologics	Time to VV-ECMO withdrawal after biologic treatment (days)	Time to IMV withdrawal after biologic treatment (days)
11 (30458534)	2019	male	41	IMV + VV-ECMO	N/A	N/A	omalizumab	4	18
5 (31136820)	2019	female	53	IMV	current smoker	N/A	reslizumab	N/A	2
6 (31485410)	2019	female	43	IMV	N/A	N/A	mepolizumab	N/A	8
7 (32938574)	2021	male	23	IMV	current smoker	childhood	benralizumab	N/A	9
8 (37357596)	2024	male	36	IMV + VV-ECMO	current smoker	childhood	reslizumab	2	20
9 (38949856)	2024	female	24	IMV + VV-ECMO	nonsmoker	childhood	benralizumab	4	5
10 (38881777)	2024	male	43	IMV + VV-ECMO	N/A	N/A	tezepelumab	7	20
Present case	2024	male	38	IMV + VV-ECMO	current smoker	childhood	tezepelumab	4	7

Abbreviations: ICU, intensive care unit; IMV, invasive mechanical ventilation; VV-ECMO, veno-venous extracorporeal membrane oxygenation; N/A, not applicable.

In conclusion, a patient with near fatal asthma that was refractory to high-dose corticosteroid therapy was successfully treated with tezepelumab in combination with IMV and VV-ECMO. This case highlights the potential role of tezepelumab as a rescue therapy for near fatal asthma in an ICU setting. Biologic agents such as tezepelumab may serve as a valuable addition to the therapeutic armamentarium for managing severe, corticosteroid-resistant asthma exacerbations.

## CRediT authorship contribution statement

**Mako Nakajima:** Data curation, Writing – original draft. **Masashi Matsuyama:** Conceptualization, Data curation, Formal analysis, Writing – original draft, Writing – review & editing. **Yuki Yamazaki:** Data curation. **Hiroya Sunabe:** Data curation. **Yuta Takahashi:** Data curation. **Keishun Boku:** Data curation. **Takeshi Miura:** Data curation. **Tomohiro Tamura:** Data curation. **Yoshiaki Inoue:** Supervision, Writing – review & editing. **Nobuyuki Hizawa:** Conceptualization, Supervision, Writing – review & editing.

## Declaration of competing interest

The authors declare that they have no known competing financial interests or personal relationships that could have appeared to influence the work reported in this paper.
